# Post-Transplant Malignancies following Pancreas Transplantation: Incidence and Implications on Long-Term Outcome from a Single-Center Perspective

**DOI:** 10.3390/jcm10214810

**Published:** 2021-10-20

**Authors:** Felix J. Krendl, Franka Messner, Claudia Bösmüller, Stefan Scheidl, Benno Cardini, Thomas Resch, Annemarie Weissenbacher, Rupert Oberhuber, Manuel Maglione, Stefan Schneeberger, Dietmar Öfner, Christian Margreiter

**Affiliations:** Center for Operative Medicine, Department of Visceral, Transplant and Thoracic Surgery, Medical University of Innsbruck, 6020 Innsbruck, Austria; felix.krendl@i-med.ac.at (F.J.K.); claudia.boesmueller@tirol-kliniken.at (C.B.); stefan.scheidl@tirol-kliniken.at (S.S.); benno.cardini@i-med.ac.at (B.C.); thomas.resch@i-med.ac.at (T.R.); annemarie.weissenbacher@i-med.ac.at (A.W.); rupert.oberhuber@i-med.ac.at (R.O.); manuel.maglione@i-med.ac.at (M.M.); stefan.schneeberger@i-med.ac.at (S.S.); dietmar.oefner@i-med.ac.at (D.Ö.); christian.margreiter@tirol-kliniken.at (C.M.)

**Keywords:** graft survival, immunosuppression, incidence, malignancy, pancreas transplantation

## Abstract

Chronic immunosuppression is associated with an increased risk of malignancy. The main objective of this study is to evaluate the incidence and effect of post-transplant malignancies (PTMs) following pancreas transplantation. The 348 first pancreas transplants performed between 1985 and 2015 were retrospectively analyzed in this study. Incidences of PTMs, as well as patient and graft survival, were evaluated. Out of 348 patients, 71 (20.4%) developed a PTM. Median time to diagnosis was 130 months. Thirty-six patients (50.7%) developed skin cancers (four patients with melanoma, 32 with NMSCs). Solid organ malignancy occurred in 25 (35.2%), hematologic malignancy in ten patients (14.1%). Affected patients were transplanted earlier [2000 (IQR 1993−2004) vs. 2003 (IQR 1999−2008); *p* < 0.001]. No differences in induction therapy were seen, both groups demonstrated comparable patient and graft survival. Pancreas transplant recipients with solid organ and hematologic malignancies had a three- and six-fold increased hazard of death compared to those with skin cancers [aHR 3.04 (IQR 1.17–7.91); *p* = 0.023; aHR 6.07 (IQR 1.87–19.71); *p* = 0.003]. PTMs affect every fifth patient following pancreas transplantation. Skin cancers are the most common malignancies accounting for 50% of all PTMs. These results underscore the importance of close dermatologic follow-up.

## 1. Introduction

Since the first pancreas transplantation in 1966, outcomes have improved consistently due to technical advances, meticulous pretransplant recipient evaluation, and new immunosuppressive regimens [1,2,3,4]. Today, five-year patient and graft survival rates are 93% and 75% for simultaneous pancreas kidney (SPK) transplantation, respectively, and even long-term pancreas graft survival of 20 years and more has been reported [5,6]. Long-term graft survival also means long-term immunosuppressive therapy for the transplant recipient. Solid organ transplant recipients (SOTRs) who receive chronic immunosuppressive therapy are at an increased risk to develop malignancies [7,8,9,10,11,12,13]. It is estimated that SOTRs have a two- to four-fold increased risk to develop PTMs compared to the general population [8,10,14,15,16].

Non-melanoma skin cancers (NMSCs), as well as post-transplant lymphoproliferative disorders (PTLDs,) are especially prevalent among SOTRs [11,15]. Pancreas transplant recipients require higher doses of immunosuppression compared to kidney or liver transplant recipients and induction therapy with T-cell depleting antibodies is routinely used nowadays in the setting of pancreas transplantation [17]. The duration and intensity of immunosuppressive regimens are associated with a higher risk of de novo malignancy following transplantation [13,18,19]. Therefore, pancreas transplant recipients may be especially vulnerable regarding the development of post-transplant malignancies (PTMs). While previous studies have mostly focused on outcomes following kidney and other solid organ transplantation [12,14,15,20], data concerning PTMs in the context of pancreas transplantation are scarce [21,22,23,24,25].

The aim of this paper is to evaluate the incidence, characteristics and potential risk factors of PTMs in pancreas transplant recipients in a European single-center. Additionally, the influence of PTMs on patient and graft survival following pancreas transplantation are analyzed.

## 2. Materials and Methods

### 2.1. Study Population

The study was approved by the ethics board of the Medical University of Innsbruck (No. 1199/2020). At the Medical University of Innsbruck 484 consecutive first pancreas transplants performed between 1st January 1985 and 31 December 2015 were evaluated for this study. After exclusion of patients lost to follow-up [*n* = 46 (9.5%)] and patients with incomplete follow-up data [*n* = 90 (18.6%)], 348 patients were included in the final analysis. Of those 348 patients, 333 received a SPK transplant, eight patients a pancreas after kidney (PAK) and seven patients a pancreas transplant alone (PTA).

### 2.2. Surgical Technique

Donor: Pancreas graft procurement was carried out as previously described elsewhere [26]. All grafts were retrieved in a no-touch technique after perfusion with Eurocollins, University of Wisconsin, or histidine-tryptophan-ketoglutarate solution. Back-table preparation of the pancreas graft included reconstruction of the mesenteric and splenic artery using the iliac bifurcation (Y-graft) from the same donor.

Recipient: Pancreas transplantation was carried out according to standard techniques [1,27]. The kidney graft was positioned retroperitoneally into the left iliac fossa and anastomosed to the external iliac vein and artery. The pancreas graft was positioned intra-abdominally in the right lower quadrant. The portal vein was anastomosed to the inferior vena cava or to the superior mesenteric vein, the Y-graft to the right common iliac artery. The technique for exocrine drainage changed over time. Since 1997 enteric diversion via side-to-side anastomosis of the duodenal segment to the proximal jejunum (40 cm distal to ligament of Treitz) is routinely performed. Prior to 1997, in case of segmental pancreas graft transplantation, exocrine drainage was achieved using duct occlusion (Ethibloc, Ethicon^®^), delayed duct occlusion, Roux-en-Y reconstruction or bladder drainage [1,27,28]. In case of bladder drainage, the portal vein was anastomosed to the right common or right external iliac vein (systemic-bladder drainage).

### 2.3. Immunosuppression and Postoperative Care

Since 1997 the standard immunosuppressive regimen for pancreas transplant recipients at our center consisted of the following: Induction therapy with a T-cell depleting agent (Alemtuzumab 30 mg, Thymoglobuline 4 mg/kg bodyweight or Anti-thymocyte globulin 8 mg/kg bodyweight respectively; dosages according to center protocol) combined with an intra-operative bolus of 500 mg methylprednisolone. Post-operatively, patients received tacrolimus (Tac) (initial trough levels 12–14 ng/mL, gradually decreased to 8 ng/mL at 6 months, 6 ng/mL at 12 months, and 4–6 ng/mL thereafter) and either mycophenolate mofetil (MMF) (1000 mg twice daily) or mycophenolic acid (MPA) (720 mg twice daily). Steroids were gradually tapered to 5 mg prednisolone per day as part of the maintenance therapy. Complete steroid withdrawal was considered on an individual basis considering the side effect profile as well as the patient’s immunologic risk. Reasons to divert from our standard protocol were mostly related to recipient factors. Conversion from Tac to Cyclosporine A (CsA) was considered in case of long-QT syndrome, or tacrolimus-associated neurotoxicity (initial trough levels 200–220 ng/mL, gradually decreased to 130–150 ng/mL at 6 months, 100 ng/mL at 12 months and 60–80 ng/mL thereafter). MMF/MPA was switched to Azathioprine (Aza) in case of gastrointestinal side effects or to avoid the teratogenic potential in patients wishing to become pregnant. In case a patient developed a PTM, immunosuppression was usually reduced based on the patient’s immunologic risk factors. In case of NMSCs, patients were preferably converted from either Tac or MMF/MPA (based on the patient’s leukocyte count) to a mTOR inhibitor. In case of all other PTMs, the maintenance immunosuppression was reduced to Tac/CsA trough levels of 3–4 ng/mL and 30–50 ng/mL, respectively, while also reducing MMF/MPA dosing by 50 %. In patients with chemotherapy-associated leukopenia MMF/MPA or Aza were discontinued. In case of severe leukopenia during chemotherapy (leukocyte count < 2.0 × 10^9^/L) steroid monotherapy (15 to 20 mg based on patient’s bodyweight) was administered. Prior to 1997 antibody induction therapy was not routinely administered. All patients received steroids as part of the induction and maintenance therapy as well as CsA combined with either Aza or MMF. Routine antimicrobial prophylaxis consisted of tazobactam/piperacillin, ciprofloxacin, and fluconazole. Trimethoprim-sulfamethoxazole and valganciclovir prophylaxis was given in all patients except those matched (Donor-/Recipient-) for 3 to 6 months. Somatostatin was administered for a total of 10 days postoperatively, and an IV heparin drip for the first 5 postoperative days was also part of the standard protocol. Blood glucose levels were kept below 150 mg/dL in the ICU. In the general ward, levels exceeding 180 mg/dL were treated with subcutaneous insulin. Grafts were monitored closely by ultrasound examination.

### 2.4. Definitions

Post-transplant malignancy was defined as any newly diagnosed primary cancer diagnosed not earlier than 90 days following pancreas transplantation. Subsequent secondary cancers were recorded but not included in the incidence analysis.

### 2.5. Outcomes

Primary outcome was the incidence of post-transplant malignancies following pancreas transplantation. Secondary outcomes included patient and graft survival.

### 2.6. Statistical Analysis

Chi-square tests and rank-sum tests were applied as appropriate. Kaplan–Meier-Plots and log-rank test, as well as Cox proportional hazard regression adjusted for donor sex, recipient age, year of transplant, and exocrine drainage were used to analyze patient and graft survival. A two-sided *p*-value ≤ 0.05 was considered statistically significant. Results were reported as frequencies and percentages for counts and medians with interquartile range for continuous variables. Risks were reported as hazard ratios with 95% confidence intervals. Statistical analysis was performed using SPSS Statistics (IBM) Version 26 for Mac. Figures were created using SPSS Statistics (IBM) Version 26 for Mac.

## 3. Results

### 3.1. Incidence and Types of Post-Transplant Malignancies

Of the 348 patients included in the study, 71 (20.4%) developed a PTM. Overall, 5-, 10-, 15-, and 25-year incidence rates were 2.0%, 8.6%, 14.9% and 20.1 %, respectively (Table 1). Thirty-six patients (50.7%) developed skin cancers. Solid organ malignancy occurred in 25 (35.2%), hematologic malignancy in 10 patients (14.1%). Of the 36 patients with skin cancers, 4 (11.1%) developed melanoma, while 32 (89.9%) developed NMSCs. Common solid organ malignancies were lung cancers with 6 patients (24%) developing non-small cell lung cancer, prostate cancer [*n* = 3 (12%)] and renal cell carcinoma [*n* = 3 (12%)]. Out of 10 patients developing hematologic malignancies, 6 (60%) developed PTLDs with an overall cumulative PTLD incidence of 1.7% (6 out of 348). One PTLD occurred within the first two years of transplantation (early PTLD), while the other five PTLDs occurred more than two years after transplantation (late PTLDs). Three PTLDs were Epstein–Barr virus (EBV)-associated (50%) including the only observed early PTLD. Nineteen patients (26.8%) developed a second, subsequent malignancy (Supplementary Table S1).

### 3.2. Donor and Recipient Demographics

Median donor age [median 27 years (IQR 18–36) vs. 28 years (IQR21–40); *p* = 0.172], donor BMI [median 23.1 kg/m2 (IQR 21.9–24.7) vs. 23.4 (IQR 21.6–24.7); *p* 0.764] and PDRI [median 0.89 (IQR 0,82–1,33) vs. 1.05 (0.85–1.40); *p* = 0.124] did not differ between patients who went on to develop a PTM and those who did not. However, patients receiving a pancreas graft from a male donor were more likely to develop a PTM [*n* = 58 (81.7%) vs. 173 (62.5%); *p* = 0.002] (Table 2).

Recipients with PTMs were transplanted earlier compared to those without PTMs [median year of transplantation 2000 (IQR 1993–2004) vs. 2003 (1999–2008); *p* < 0.001]. Type of exocrine drainage [enteric *n* = 48 (67.6%) vs. 240 (86.6%), bladder *n* = 18 (25.4%) vs. 35 (12.6%); *p* < 0.001] did also differ between groups with and without PTMs. Type of transplantation [SPK *n* = 67 (94.4%) vs. 266 (96%), PAK *n* = 1 (1.4%) vs. 7 (2.5%) and PTA *n* = 3 (4.2%) vs. 4 (1.4%); *p* = 0.287], recipient age [median 44 years (IQR 36–51) vs. 41 years (IQR 34–48); *p* = 0.058], recipient BMI [median 23.1 (IQR 21,8–26,0) vs. 23.1 (IQR 21.1–25.2); *p* = 0.767], recipient sex [female *n* = 20 (28.2%) vs. 105 (37.9%); *p* = 0.165], use of induction therapy [*n* = 48 (67.6%) vs. 205 (74%); *p* = 0.280] and type of endocrine drainage [systemic *n* = 61 (85.9%) vs. 250 (90.3%), portal *n* = 4 (5.6%) vs. 20 (7.2%); *p* > 0.9] were similar between groups (Table 3).

### 3.3. Time to Malignancy

**Figure 1:**
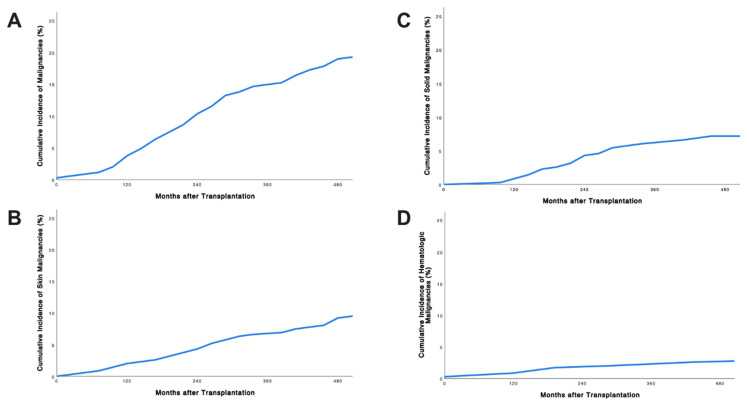
Overall cumulative incidence (**A**) as well as cumulative incidence of skin (**B**), solid (**C**), and hematologic (**D**) post-transplant malignancies (PTMs) after pancreas transplantation is shown. The median time to malignancy was 130 months and similar between all types of PTMs (log-rank *p* = 0.125).

Median age at diagnosis was 55 years (IQR 50–61 years). Overall, median time to diagnosis was 130 months (IQR 90–206 months). Median time to diagnosis for skin cancers, solid organ malignancies, and hematologic malignancies was 142 months (IQR 123–161), 121 months (IQR 104–138) and 92 months (IQR 51–133), respectively, and was similar between groups (*p* = 0.125). The first skin cancer was diagnosed 24 months following transplantation, the first solid organ malignancy 49 months after transplantation and the first hematologic malignancy, an EBV-associated PTLD, 6 months after transplantation (Figure 1).

### 3.4. Patient and Graft Survival

**Figure 2:**
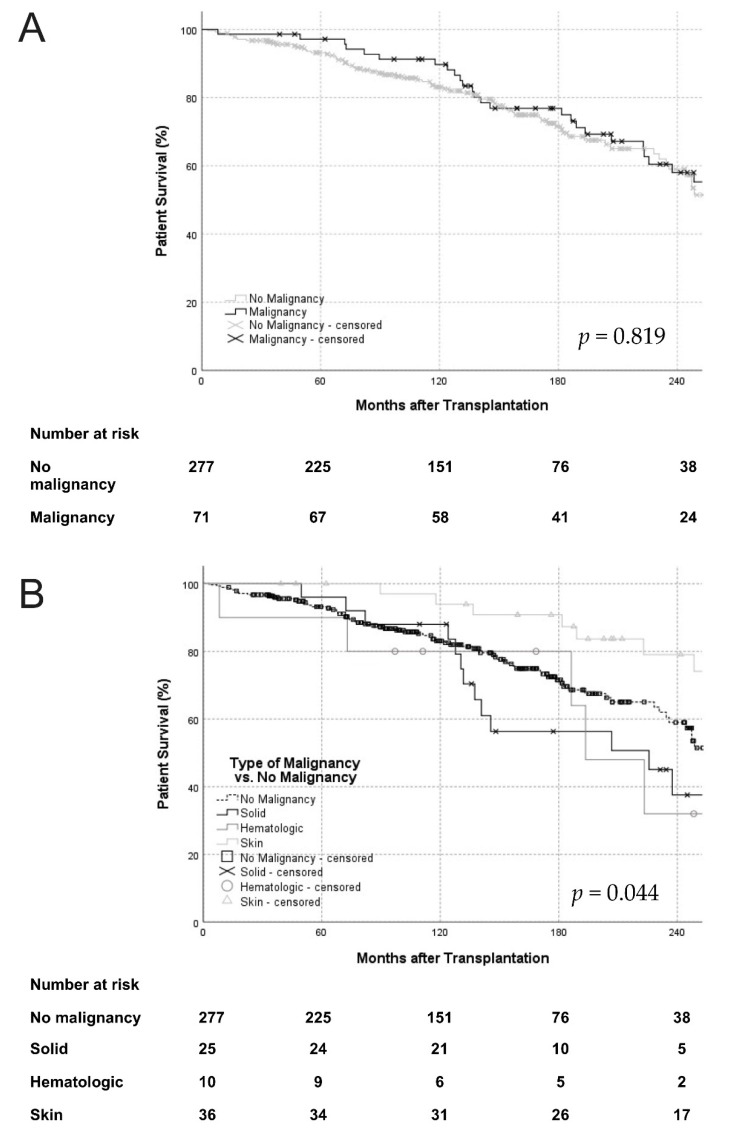
Overall patient survival (**A**) and patient survival based on the type of PTM (**B**). Overall patient survival was similar between patients with and without PTM (log-rank *p* = 0.819) but significantly different when stratified according to the type of PTM (log-rank *p* = 0.044). *PTMs, post-transplant malignancies*.

Similar patient survival was observed in patients with and without PTMs. However, a subgroup analysis showed that differences in patient survival did exist between different types of malignancy (Figure 2). Pancreas transplant recipients who developed solid organ or hematologic malignancies had an increased hazard of death when compared to pancreas transplant recipients who developed skin cancers [solid organ malignancy aHR 3.04 (95% CI 1.17–7.91); *p* = 0.023, hematologic malignancy aHR 6.07 (95% CI 1.87–19.71); *p* = 0.003]. Patients with post-transplant skin cancers had a decreased hazard of death compared to patients without PTMs [aHR 0.37 (95% CI 0.18–0.76); *p* = 0.007]. Patients with solid organ and hematologic malignancies had similar hazards of death compared to patients without malignancy [solid organ malignancy aHR 0.97 (95% CI 0.52–1.79); *p* = 0.914, hematologic malignancy aHR 1.77 (95% CI 0.76–4.14); *p* = 0.168] (Figure 2). Overall, cardiac events [*n* = 36 (34.6%)] were responsible for most deaths, followed by infection [*n* = 31 (29.8%)] and malignancy [*n* = 15 (14.4%)].

**Figure 3:**
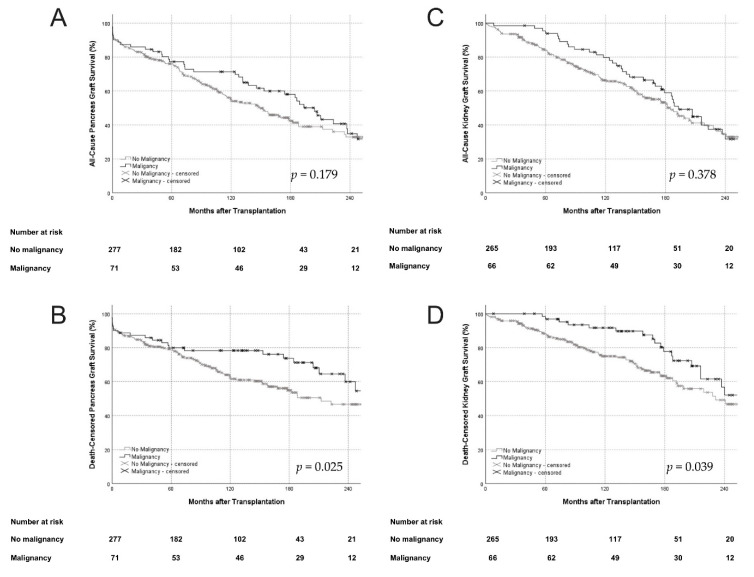
All-cause (**A**,**C**) and death censored (**B**,**D**) pancreas and kidney graft survival. While all-cause pancreas and kidney graft survival were similar between groups, death censored pancreas and kidney graft survival were significantly superior in patients with PTMs. PTMs, post-transplant malignancies.

All-cause pancreas and kidney graft survival were comparable between groups. Death censored pancreas and kidney graft survival was significantly higher in patients with PTMs (Figure 3A or Figure 3B). At 10 years post-transplant, all-cause and death-censored pancreas and kidney graft survival in patients with and without PTMs were 66.7% and 53.4%, 78.6% and 61.6%, as well as 79.6% and 66.3%, and 91.7% and 75.0%. All-cause as well as death censored pancreas and kidney graft survival was similar when stratified according to subtype of PTMs (Supplementary Figures S1 and S2). Out of 71 patients who developed a PTM, 43 patients had lost their pancreas graft at last follow-up. Pancreas graft loss following a PTM diagnosis (potential graft loss related to malignancy) occurred in 24 patients (55.8%) (Supplementary Table S2).

## 4. Discussion

The present study investigated the incidence of PTMs following pancreas transplantation in a European single-center. Our analysis demonstrated that PTMs affect every 5th patient after pancreas transplantation. Patient- as well as all-cause pancreas and kidney graft survival rates were similar between patients with and without PTMs. While patients with skin malignancies showed a superior patient survival compared to patients without malignancies, patients with solid and hematologic cancers demonstrated a similar survival. Only when comparing the latter to patients with skin cancers a significantly inferior patient survival was seen. Our data did not show any association between induction therapy and the development of PTM. Patients who developed a malignancy, however, were transplanted earlier in time and were significantly longer alive with a functioning graft. This is underscored by the observation that patients who went on to develop a PTM were more likely to have received a bladder drainage, a technique that significantly correlated with year of transplantation (*p* < 0.001) in our dataset and that was more commonly performed in the earlier years of pancreas transplantation at our center. Time under immunosuppression is known to be an independent risk factor for the development of PTMs after SOT [8,13]. This is in accordance with our findings, indicating that the cumulative immunosuppressive burden is the most important single factor contributing to the development of PTMs.

Similarly to our results, Wimmer et al. did not find an association between induction therapy and PTMs [8]. However, in a subgroup analysis, an increased risk of PTLDs in patients with T-cell depleting antibody induction was seen [8]. Opelz et al. also found T-cell depleting antibodies but not IL-2 antibodies to be a risk factor for the development of PTLDs [29].

Our analysis showed an overall PTM incidence of 20.4% following pancreas transplantation. Previous single-center studies have reported incidence rates between 6% and 8% after pancreas transplantation [21,22,23]. The observed differences compared to our results might be attributed to the fact that NMSCs were not reported and that the median follow-up time was significantly shorter. Ethnic differences may also have played a role since Tomimaru et al. didn’t observe any post-transplant skin cancers when analyzing a Japanese cohort, which is in accordance with the fact that skin malignancies are extremely rare in Japanese transplant recipients [21,30,31]. When excluding skin cancers, the incidence of PTMs in our cohort was 10.1% and thus similar to the incidence rates reported by others [21,22].

Wimmer et al. analyzed 2419 kidney transplant recipients in a German transplant center with a median follow-up time of 9.5 years and found an overall PTM incidence of 21%, which was similar to the overall incidence of 20.4% we found in our analysis [8]. This is an interesting finding since pancreas transplant recipients usually require a more intense immunosuppressive regimen compared to kidney transplant recipients. So, one would expect the PTM incidence rate to be higher in a pancreas transplant cohort. In a large study from the UK including all SOTRs 15% of patients developed a PTM [15].

Skin cancers were the most common cancers accounting for 50% of all PTMs in our cohort. This is in line with previous reports in the literature where skin cancers are found to be responsible for 40–50% of all PTMs after SOT, with 90–95% of these skin cancers being NMSCs [10,13,15,32,33,34]. In the general population basal cell carcinomas (BCCs) are more common than squamous cell carcinomas (SCCs). In SOTRs however, this ratio is reversed, and SCCs are more common than BCCs [35]. Human papillomavirus (HPV) seems to play a role in SCC but not BCC pathogenesis [36,37]. Since SOTRs, like HIV positive patients, are more susceptible to oncogenic viruses [38,39], this might explain why SCCs are more common in SOTRs than in the general population.

EBV, another oncogenic virus, is implicated in the pathogenesis of PTLDs. The overall incidence of PTLDs after SOT lies between 1% and 20% based on the transplanted organ as well as type and intensity of immunosuppression [40]. The cumulative five-year incidence of PTLDs after pancreas transplantation has been reported to range from 1.0% to 2.5% [41,42]. In our cohort, the cumulative incidence of PTLDs after 25 years was 1.7% and thus lower than in previous reports [43].

Our study demonstrated that the development of PTMs per se did not negatively influence patient survival. We could, however, observe differences between different kinds of malignancies. To our knowledge, this is the first study investigating the incidence and outcomes of PTMs following pancreas transplantation based on the type of malignancy. Patients with skin cancers had an improved survival compared to patients without malignancies. This might be due to the fact that the majority of skin cancers (89%) were NMSCs which generally carry a good prognosis if detected early. Five- and 10-year survival rates close to 100% have been reported for patients with BCCs [44]. Eisemann et al. analyzed NMSC patients in a large German population study and found a relative five-year survival of 102.9% for patients with BCCs, meaning that patients with BCCs actually had a survival advantage compared to the general population [45]. The authors credited the survival advantage to health-promoting factors related to the BCC diagnosis, such as changes to a healthier lifestyle. When comparing patients with SCCs to the general population, Eisemann et al. found a relative five-year survival rate of 93.6% [45]. Therefore, it seems plausible that a diagnosis of NMSC did not negatively impact overall survival in our analysis and even conferred a survival advantage compared to patients without malignancies. When comparing patients with solid and hematologic malignancies to patients with skin cancers, we found an increased hazard of death for those patients. PTLDs are known to have a poor prognosis, with 40% to 50% of patients dying within the first year of diagnosis [29]. However, these figures seem to have improved recently with modern therapy concepts including rituximab, chemotherapy, and reduction of immunosuppression [46].

At first sight, our findings might seem to contradict previously published studies describing an excess cancer mortality in SOTRs [47,48,49]. However, most of these studies compare cancer mortality in SOTRs with the general population and thus are not comparable to our analysis where hazard of death was compared in SOTRs with and without PTMs. Fröhlich et al. recently presented data comparing patient survival in patients with and without PTMs after kidney transplantation, similar to our analysis [50]. The authors found a significantly worse survival in the malignancy group. However, NMSCs were excluded, and only solid as well as hematologic tumors were assessed. These entities are known to portend worse outcomes compared to NMSCs.

The limitations of this study are mostly due to the retrospective single center design of the analysis. Detailed information on changes of immunosuppressive regimen once patients had been discharged from the hospital is lacking. This is because not all patients were followed at our center and thus, not every change in immunosuppressive therapy over the substantial follow-up period was accurately captured by this retrospective analysis. The observation that patients with NMSCs showed better overall survival compared to patients without a PTM may reflect a selection bias. Patients with functioning grafts may experience better overall survival and, thus, might have a higher cumulative risk to develop malignancies. The longer estimated median kidney and pancreas graft survival in patients with PTMs compared to those without (PTM: kidney 176 months [IQR 118–223], pancreas 153 months [IQR 58–212]; No PTM: kidney 107 months (IQR (57–168), pancreas 94 months (IQR 36–159); *p* < 0.001) is possibly attributed to the longer overall follow-up time in patients with PTMs. Strengths of this analysis are the relatively large sample size of pancreas transplant recipients, the long-observation period and a long follow-up time compared to other published reports on the subject.

## 5. Conclusions

PTMs affect every fifth patient following pancreas transplantation. Skin cancers are the most common malignancies accounting for 50% of all PTMs. To our knowledge, this is the first study reporting outcomes of PTMs following pancreas transplantation based on type of malignancy. Interestingly, patients with NMSC demonstrated superior survival, a finding that has previously been described in the general population. These results underscore the importance of close dermatologic follow-up after pancreas transplantation and SOT in general.

## Figures and Tables

**Table 1:** Overall and cumulative incidence of PTMs over time.

All Patients Patients with PTMs			*N* = 348 *n* = 71	
Overall Incidence: 20.4%				
Cumulative Incidence Over Time				
	5-years	10-years	15-years	25-years
All PTMs	2.0	8.6	14.7	20.1
Skin	1.4	3.7	6.6	10.1
NMSC	0.9	2.6	3.7	6.9
Solid	0.3	3.2	6.0	7.2
Hematologic	0.3	1.7	2.0	2.9
PTLD	0.3	1.1	1.4	1.7

Values are presented as percentages unless indicated otherwise. NMSC, non-melanoma skin cancer; PTLD, post-transplant lymphoproliferative disorder; PTMs, post-transplant malignancies.

**Table 2:** Donor characteristics.

	All	PTM	No PTM	*p*-Value
Age	28 (20–39)	27 (18–36)	28 (21–40)	0.172
BMI	23.3 (21.6–24.7)	23.1 (21.9–24.7)	23.4 (21.6–24.7)	0.764
COD				0.389
Trauma	186 (53.4)	45 (63.4)	141 (50.9)	
CVA	87 (25)	15 (21.1)	72 (26)	
SAH	41 (11.8)	6 (8.5)	35 (12.6)	
Suicide	25 (7.2)	3 (4.2)	22 (7.9)	
Other	9 (2.6)	2 (2.8)	7 (2.5)	
Sex (female)	117 (33.6)	13 (18.3)	104 (37.5)	0.002
PDRI	1.04 (0.84–1.38)	0.98 (0.82–1.33)	1.05 (0.85–1.40)	0.124

Values are presented as medians or absolute numbers with IQRs and percentages in parentheses. BMI, body mass index; COD, cause of death; CVA, cerebrovascular accident; IQR, interquartile range; PDRI, pancreas donor risk index; PTM, post-transplant malignancies; SAB, subarachnoid hemorrhage.

**Table 3:** Recipient characteristics.

	All	PTM	No PTM	*p*-Value
Age	41 (35–49)	44 (36–51)	41 (34–48)	0.058
BMI	23.1 (21.3–25.2)	23.1 (21.8–26.0)	23.1 (21.1–25.2)	0.767
Sex (female)	125 (35.9)	20 (28.2)	105 (37.9)	0.165
Tx year	2003 (1998–2007)	2000 (1993–2004)	2003 (1999–2008)	<0.001
Waitlist time (months)	5.4 (2.4–9.9)	4.6 (1.9–8.9)	5.7 (2.4–10.1)	0.225
Tx type				0.287
SPK	333 (95.7)	67 (94.4)	266 (96.0)	
PAK	8 (2.3)	1 (1.4)	7 (2.5)	
PTA	7 (2.0)	3 (4.2)	4 (1.4)	
Endocrine drainage				>0.9
Systemic	311 (89.4)	61 (85.9)	250 (90.3)	
Portal	24 (6)	4 (5.6)	20 (7.2)	
Missing	13 (3.7)	6 (8.5)	7 (2.5)	
Exocrine drainage				<0.001
Enteric	288 (82.8)	48 (67.6)	240 (86.6)	
Bladder	53 (15.2)	18 (25.4)	35 (12.6)	
Other	4 (1.1)	3 (4.2)	1 (0.4)	
Missing	3 (0.9)	2 (2.8)	1 (0.4)	
DM type				>0.9
Type I	333 (95.7)	68 (95.8)	265 (95.7)	
Type II	15 (4.3)	3 (4.2)	12 (4.3)	
CMV status				0.904
D + /R +	75 (21.6)	14 (19.7)	61 (22.0)	
D + /R −	74 (21.3)	11 (15.5)	63 (22.7)	
D − /R +	61 (17.5)	10 (14.1)	51 (18.4)	
D − /R −	62 (17.8)	9 (12.7)	53 (19.1)	
Missing	76 (21.8)	27 (38.0)	49 (17.7)	
COD				<0.001
Cardiac	36 (34.6)	4 (12.9)	32 (43.8)	
Infectious	31 (29.8)	8 (25.8)	23 (31.5)	
CVA	9 (8.7)	1 (3.2)	8 (11.0)	
Hemorrhage	5 (4.8)	0 (0.0)	5 (6.8)	
PTM	15 (14.4)	15 (48.4)	-	
Other	8 (7.7)	3 (9.7)	5 (6.8)	
Follow-up time (months)	145 (84–206)	195 (132–262)	134 (75–184)	<0.001

Values are presented as medians or absolute numbers with IQRs and percentages in parentheses. BMI, body mass index; CMV, cytomegalovirus; COD, cause of death; CVA, cerebrovascular accident; D, donor; DM, diabetes mellitus; IQR, interquartile range; PAK, pancreas after kidney; PDRI, pancreas donor risk index; PTA, pancreas transplant alone; PTM, post-transplant malignancy; SAB, subarachnoid hemorrhage; SPK, simultaneous pancreas kidney; Tx, transplant.

## Data Availability

Data are available from the corresponding author upon reasonable request.

## Supplementary Materials

The following are available online at https://www.mdpi.com/article/10.3390/jcm10214810/s1, Table S1: Incidence and types of subsequent PTMs. Table S2: Pancreas graft loss related to malignancy. Figure S1: Pancreas graft survival according to PTM subtype. Figure S2: Kidney graft survival according to PTM subtype.
