# Supplementary material for: Post-Transplant Malignancies following Pancreas Transplantation: Incidence and Implications on Long-Term Outcome from a Single-Center Perspective
Source: J Clin Med. 2021 Oct 20;10(21):4810. doi: 10.3390/jcm10214810 (PMC8584646; doi:10.3390/jcm10214810)
Supplement: Supplementary file 1 [file jcm-10-04810-s001.zip › jcm-1409084-supplementary.pdf]

Supplementary Material

Table S1. Incidence and types of subsequent PTMs.

Overall incidence	
**All patients**	N = 348
**Patients with PTMs**	71 (20.4 %)
**Patients with subsequent PTMs**	19 (5.5 %)
**Time to subsequent PTM diagnosis**	46 months
**Subsequent PTMs**	
**Skin**	8 (42.1 %)
Melanoma	2 (25 %)
NMSC	6 (75 %)
**Solid**	9 (47.4 %)
**Hematologic**	2 (10.5 %)
PTLD	1 (50 %)
Other	1 (50 %)

Values are presented as absolute numbers and percentages unless indicated otherwise. NMSC, non-melanoma skin cancer; PTLD, post-transplant lymphoproliferative disorder; PTMs, post-transplant malignancies.

Table S2. Pancreas graft loss related to malignancyc.

Overall incidence	
**Patients with PTMs**	N = 71 (100 %)
**Patients with FPG at last follow up**	28 (39.4 %)
**Patients without FPG at last follow up**	43 (60.6 %)
**PG lost before PTM diagnosis**	19 (44.2 %)
**PG lost following PTM diagnosis**	24 (55.8%)
**Cause of PGL following PTM diagnosis**	N = 24
**Unknown**	1 (4.2 %)
**Rejection**	4 (16.7 %)
**Death with FPG**	19 (79.2 %)
**Cause of death**	
Malignancy	11 (57.9 %)
Sepsis	5 (26.3 %)
Cardiac events	1 (5.3 %)
Pulmonary embolus	1 (5.3 %)
Unspecified	1 (5.3 %)

Values are presented as absolute numbers and percentages unless indicated otherwise. FPG, functioning pancreas graft; PG, pancreas graft; PGL, pancreas graft loss; PTMs, post-transplant malignancies.

A

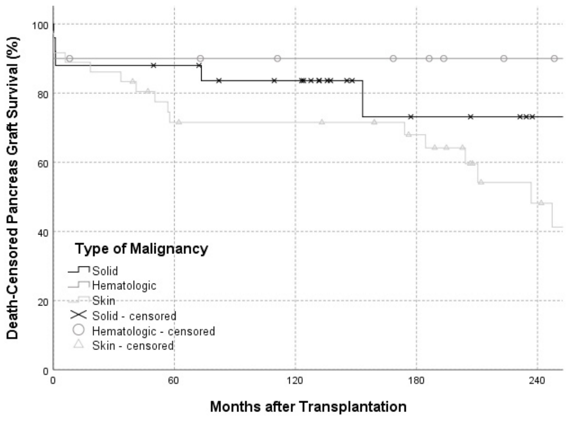

B

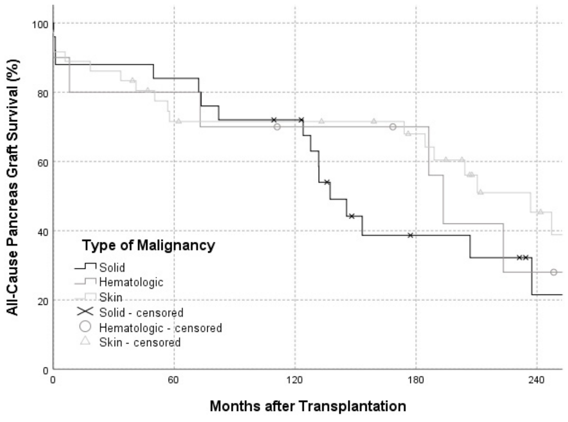

Number at risk					
Solid	25	21	17	6	2
Hematologic	10	8	6	5	2
Skin	36	24	23	18	8

C

Pancreas	aHR	95%CI	P-value
ACGF			
Skin	Ref.	Ref.	
Solid	1,61	0,77-3,36	0,207
Hematologic	1,80	0,70-4,63	0,225
DCGF			
Skin	Ref.	Ref.	
Solid	0,63	0,22-1,83	0,394
Hematologic	0,29	0,04-2,32	0,244
* Model adjusted for donor sex, recipient age, transplant years, exocrine drainage			

Figure S1. Pancreas graft survival according to PTM subtype. Death censored (A) and all cause pancreas graft survival (B) comparing subtypes of PTMs. Adjusted hazard ratios for all cause and death censored pancreas graft survival (C). Comparable pancreas graft was observed when stratifying according to subtypes of PTMs. ACGF; all cause graft function; DCGF, death censored graft function; PTMs, post-transplant malignancies.

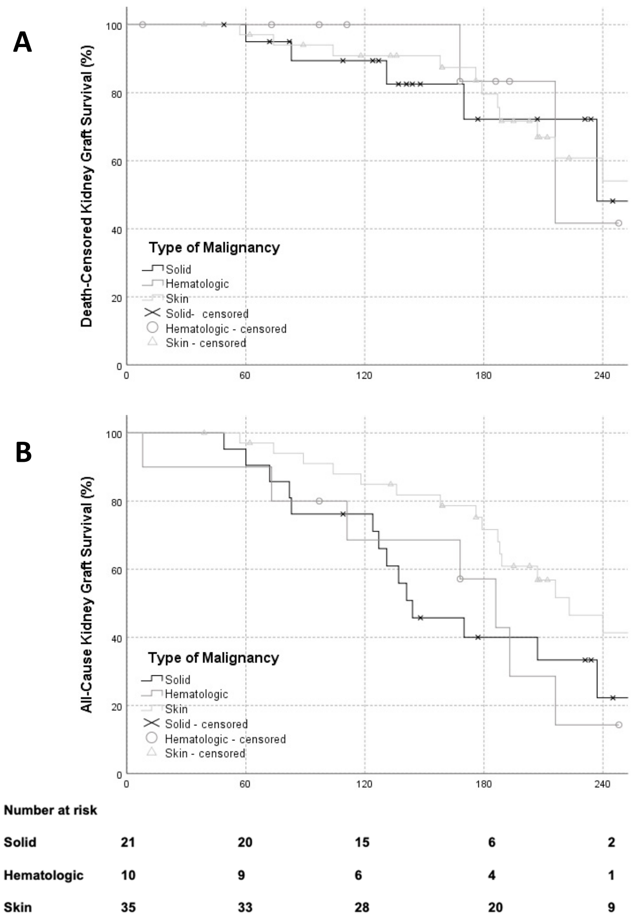

C

Kidney	aHR	95%CI	P-value
ACGF			
skin	Ref.	Ref.	
Solid	1,97	0,91-4,27	0,086
Hematologic	2,60	0,97-6,99	0,058
DCGF			
skin	Ref.	Ref.	
Solid	1,54	0,48-4,97	0,471
Hematologic	1,43	0,26-7,76	0,677

* Model adjusted for donor sex, recipient age, transplant years, exocrine drainage

Figure S2. Kidney graft survival according to PTM subtype. Death censored (A) and all cause kidney graft survival (B) comparing subtypes of PTMs. Adjusted hazard ratios for all cause and death censored kidney graft survival (C). Comparable kidney graft was observed when stratifying according to subtypes of PTMs. ACGF; all cause graft function; DCGF, death censored graft function; PTMs, post-transplant malignancies.
